# Early changes within the lymphocyte population are associated with the development of multiple organ dysfunction syndrome in trauma patients

**DOI:** 10.1186/s13054-016-1341-2

**Published:** 2016-06-07

**Authors:** Joanna Manson, Elaine Cole, Henry D. De’Ath, Paul Vulliamy, Ute Meier, Dan Pennington, Karim Brohi

**Affiliations:** Barts Centre for Trauma Sciences, Blizard Institute, QMUL, London, E1 2AT UK; Centre for Neuroscience, Blizard Institute, QMUL, London, E1 2AT UK; Centre for Immunobiology, Blizard Institute, QMUL, London, E1 2AT UK

**Keywords:** Multiple organ dysfunction syndrome, Lymphocytes, Lymphopenia, Innate immunity, Cellular immunity, Natural killer, Gamma delta T cells, Trauma, Wounds and injuries, Cytokines

## Abstract

**Background:**

Early survival following severe injury has been improved with refined resuscitation strategies. Multiple organ dysfunction syndrome (MODS) is common among this fragile group of patients leading to prolonged hospital stay and late mortality. MODS after trauma is widely attributed to dysregulated inflammation but the precise mechanics of this response and its influence on organ injury are incompletely understood. This study was conducted to investigate the relationship between early lymphocyte responses and the development of MODS during admission.

**Methods:**

During a 24-month period, trauma patients were recruited from an urban major trauma centre to an ongoing, observational cohort study. Admission blood samples were obtained within 2 h of injury and before in-hospital intervention, including blood transfusion. The study population was predominantly male with a blunt mechanism of injury. Lymphocyte subset populations including T helper, cytotoxic T cells, NK cells and γδ T cells were identified using flow cytometry. Early cytokine release and lymphocyte count during the first 7 days of admission were also examined.

**Results:**

This study demonstrated that trauma patients who developed MODS had an increased population of NK dim cells (MODS vs no MODS: 22 % vs 13 %, *p* < 0.01) and reduced γδ-low T cells (MODS vs no MODS: 0.02 (0.01–0.03) vs 0.09 (0.06–0.12) × 10^9/L, *p* < 0.01) at admission. Critically injured patients who developed MODS (*n* = 27) had higher interferon gamma (IFN-γ) concentrations at admission, compared with patients of matched injury severity and shock (*n* = 60) who did not develop MODS (MODS vs no MODS: 4.1 (1.8–9.0) vs 1.0 (0.6–1.8) pg/ml, *p* = 0.01). Lymphopenia was observed within 24 h of injury and was persistent in those who developed MODS. Patients with a lymphocyte count of 0.5 × 10^9^/L or less at 48 h, had a 45 % mortality rate.

**Conclusions:**

This study provides evidence of lymphocyte activation within 2 h of injury, as demonstrated by increased NK dim cells, reduced γδ-low T lymphocytes and high blood IFN-γ concentration. These changes are associated with the development of MODS and lymphopenia. The study reveals new opportunities for investigation to characterise the cellular response to trauma and examine its influence on recovery.

## Background

Refined resuscitation strategies have improved early survival for trauma patients [1–3]. Expediting recovery is the next challenge for clinicians as mortality after 24 h remains high and multiple organ dysfunction syndrome (MODS) is a major contributing factor [4]. MODS inflicts a substantial burden of acute and long-term morbidity upon patients requiring prolonged intensive care unit (ICU) admissions and high healthcare costs [5].

Development of MODS is widely attributed to an uncontrolled immune system dysfunction, precipitated by the release of damage-associated molecular patterns (DAMPs) from extensive tissue damage and ischaemia [6–8]. The systemic inflammatory response this generates is currently characterised by prolific release of inflammatory mediators and widespread genomic activation [6, 9–11]. High levels of inflammation correlate with worse outcomes, but the precise elements of the inflammatory process which lead to organ failure remain unclear [12].

In recent years, certain lymphocyte subsets have been identified as key components of the early, innate immune response [13–15]. This follows the discovery that they possess an intrinsic capacity for activation following direct contact with DAMPs, therefore bypassing the slower ‘adaptive’ response on which lymphocytes were previously thought to be reliant [16–18]. Lymphopenia has also been associated with increased mortality after trauma [19, 20]. Current evidence therefore suggests that lymphocytes may play an important role in the immunological response to trauma, although they have not been well characterised in the early post-injury phase.

The principal objective of this study was to describe the lymphocyte phenotype in trauma patients immediately on arrival to hospital and to assess the relationship with MODS and lymphopenia during recovery. We conducted a prospective observational cohort study at a single major trauma centre (MTC).

## Methods

### Research setting and study participants

The Royal London Hospital is an urban major trauma centre which has approximately 3000 full trauma team activations each year. A prospective observational cohort study called the ‘Activation of Coagulation and Inflammation after Trauma Study II’ (ACIT II) was established in 2008. Its purpose was to facilitate study of the biological mechanisms responsible for acute traumatic coagulopathy and the inflammatory response to trauma. It has approval from the National Health Service Research ethics committee (REC: 07/Q0603/29). All patients requiring full trauma team activation between 0800 and 2200 hours were screened for eligibility. The exclusion criteria included: age < 16 years, transfer from another hospital, arrival > 120 minutes from injury, pre-hospital administration of > 2000 ml crystalloid, > 5 % burns, severe liver disease, known bleeding abnormality (including anticoagulant medication), refused consent and vulnerable patients. Consent for incapacitated patients was initially obtained from a legally appointed representative in accordance with the Mental Health Act 2005 [21]. Written consent was requested from all participants or next of kin during the hospital stay.

### Data collection

Blood was drawn into a 3-ml EDTA vacutainer (×2) and a 4.5-ml citrated vacutainer (×2), within 10 minutes of arrival at the MTC and < 2 h from injury. All samples were taken before in-hospital interventions, such as blood transfusion or surgical procedures. Interventions prior to hospital arrival may have included intubation, mechanical ventilation, thoracostomy and external fracture splinting. Pre-hospital treatment protocols advocate minimal use of crystalloid fluid (compound sodium lactate [CSL]) except for patients in extremis. All interventions and fluid administered during the resuscitation phase were documented, in addition to demographic data. Two further blood samples were drawn at 24 h +/−1 h and on the day of 72 h. Whilst in critical care, patients had a full blood count taken daily between 0400 and 0600 hours and additional samples at the discretion of the clinical team. If patients had several blood tests within a 24-h period, the most abnormal measurements were recorded.

### Outcome measurements

Patients were reviewed daily, until death or discharge. The primary outcome measures were MODS and lymphocyte count. MODS was defined as a Sequential Organ Failure Score (SOFA) of 6 or more, on two or more consecutive days, at least 48 h after admission [4, 22–24]. Secondary outcome measures included 28-day mortality and the development of infection. Infection was defined clinically using CDC criteria and was determined by consensus between members of the research team (JM, EC) [14, 15, 25].

### Experiment methodology

#### Lymphocyte count

A differential white cell count was performed by the hospital laboratory staff, using EDTA blood samples and a Sysmex SE2100 Analyser (Sysmex, Milton Keynes, UK). Normal range for our laboratory was 1.0–4.0 × 10^9^/L and lymphopenia was therefore defined as a lymphocyte count < 1.0 × 10^9/L.

#### Flow cytometry

Freshly drawn blood from an EDTA vacutainer was gently agitated to mix the contents, then 500 ul of whole blood was withdrawn and placed in a falcon tube. Seven millilitres of warmed (37 °C) BD lysis buffer (Cat No: 552052; BD, Oxford, UK) was added and the tube briefly vortexed to achieve red cell lysis. The mixture was diluted with phosphate-buffered saline (PBS) and centrifuged at 200 g for 10 minutes. The supernatant was discarded and the cellular pellet re-suspended in the residual fluid (approximately 200 ul). Fc blocker was added and the tube incubated in the dark for 10 minutes at room temperature (RT). Titrated volumes of colour-labelled antibodies (eBioscience, San Diego, CA, USA) were then added to the cell suspension: CD45 PerCP-Cy5.5 (45–0459), CD3 PE-Cy7 (25–0038), CD4 eFluor 450 (48–0047), CD8 APC-eFluor 780 (47–0088), CD56 APC (17–0567), δγ TCR FITC (11–9959), and CD69 PE (12–0699). The solution was incubated in the dark at RT for 15 minutes. The cells were washed with 2 ml PBS and centrifuged at 200 g for 5 minutes (×2). Then samples were fixed with 4 % paraformaldehyde and stored in the dark at 4 °C. Cytometer readings were performed within 48 h using a BD Canto II flow cytometer. Lymphocytes were identified using CD45 and side scatter.

#### ELISA

Citrated vacutainer blood samples were centrifuged at 3400 rpm for 10 minutes. The plasma supernatant was then stored in aliquots at −80 °C. Plasma cytokine analysis was performed using a Meso Scale SECTOR Imager 2400 and a 7-plex platform, in accordance with their standard protocol (Meso Scale Discovery, Rockville, MD, USA).

### Patient selection

The three reported experiments were performed sequentially, using different patient cohorts. In all experiments, patient injuries were characterised using the Injury Severity Score (ISS) and base deficit (BD) at admission. BD was used as a surrogate marker for haemorrhagic shock [26, 27]. Control patients were also recruited. These were patients who underwent full trauma team assessment but were found to have no significant injuries, defined as an ISS 0–2 and BD −2 to 2 mmol/L.

The inclusion criteria varied for each experiment. The flow cytometry experiment was conducted with sequentially recruited patients of all injury levels. The cytokine experiment used specifically defined patient characteristics, namely: a blunt mechanism of injury, ISS ≥ 25, < 500 ml CSL and no blood products prior to blood draw. These patients were identified from the available ACIT II database along with some controls. The criteria were defined a priori with the intention of obtaining two comparable groups, matched for injury severity and shock but with different outcomes. In addition, we wished to exclude the immunological influence of blood products [10]. The lymphocyte count experiment included patients admitted to the ICU to enable assessment of the significance of the lymphocyte count in the patient population at risk of MODS.

### Data analysis and statistics

Data are presented as mean (95 % confidence interval [CI]) and tested using Student’s *t* test or analysis of variance (ANOVA), unless otherwise stated. Mann-Whitney *U* tests were used for non-parametric data and Fisher’s exact test was used for categorical data. Cytokine concentrations were transformed into their natural log to enable analysis with parametric tests. Survival was assessed using a Kaplan-Meier analysis and a log-rank test. Flow cytometry data was analysed using Flow Jo Software version 10.6 (Treestar, Inc., Ashland, OR, USA). Data analysis and statistics were performed using GraphPad Prism 5.01 (GraphPad, Software, Inc., La Jolla, CA, USA, Excel (Microsoft Corp., Redmond, WA, USA) or IBM SPSS version 23 (IBM Corp., Armonk, NY, USA). A *p* value of < 0.05 was considered statistically significant.

Binary logistic regression was performed, using SPSS, on the ICU cohort data (*n* = 280) to identify variables that were independently associated with MODS development. The variables entered included demographics, injury characteristics and the lymphocyte count at 48 h. Univariate analysis was conducted initially and variables identified as significant with a *p* < 0.1 were then added into a forwards likelihood-ratio stepwise regression with significance set at *p* < 0.05 for inclusion and *p* > 0.1 for removal. Goodness of fit was assessed using Hosmer-Lemeshow, Cox and Snell and Nagelkerke tests. Model variables were analysed for multicollinearity and no interdependence between the entered variables was identified as tolerance statistics were above 0.1 and variance inflation factors (VIF) were less than 10.

The 7-day trend of lymphocyte count was tested using a two-way mixed ANOVA. Data were first transformed in to their natural log to reduce variance as demonstrated by Levene's test. Despite this, several of the key assumptions required for a valid ANOVA were violated; namely, normal distribution, homogeneity of variance and sphericity. Simple main effects were therefore also tested, at each time point, using general linear model univariate analysis.

## Results

This study was conducted over a 24-month period with three separate patient cohorts. The demographics are presented separately.

### Early changes in lymphocyte subpopulations are associated with the development of MODS

**Fig. 1:**
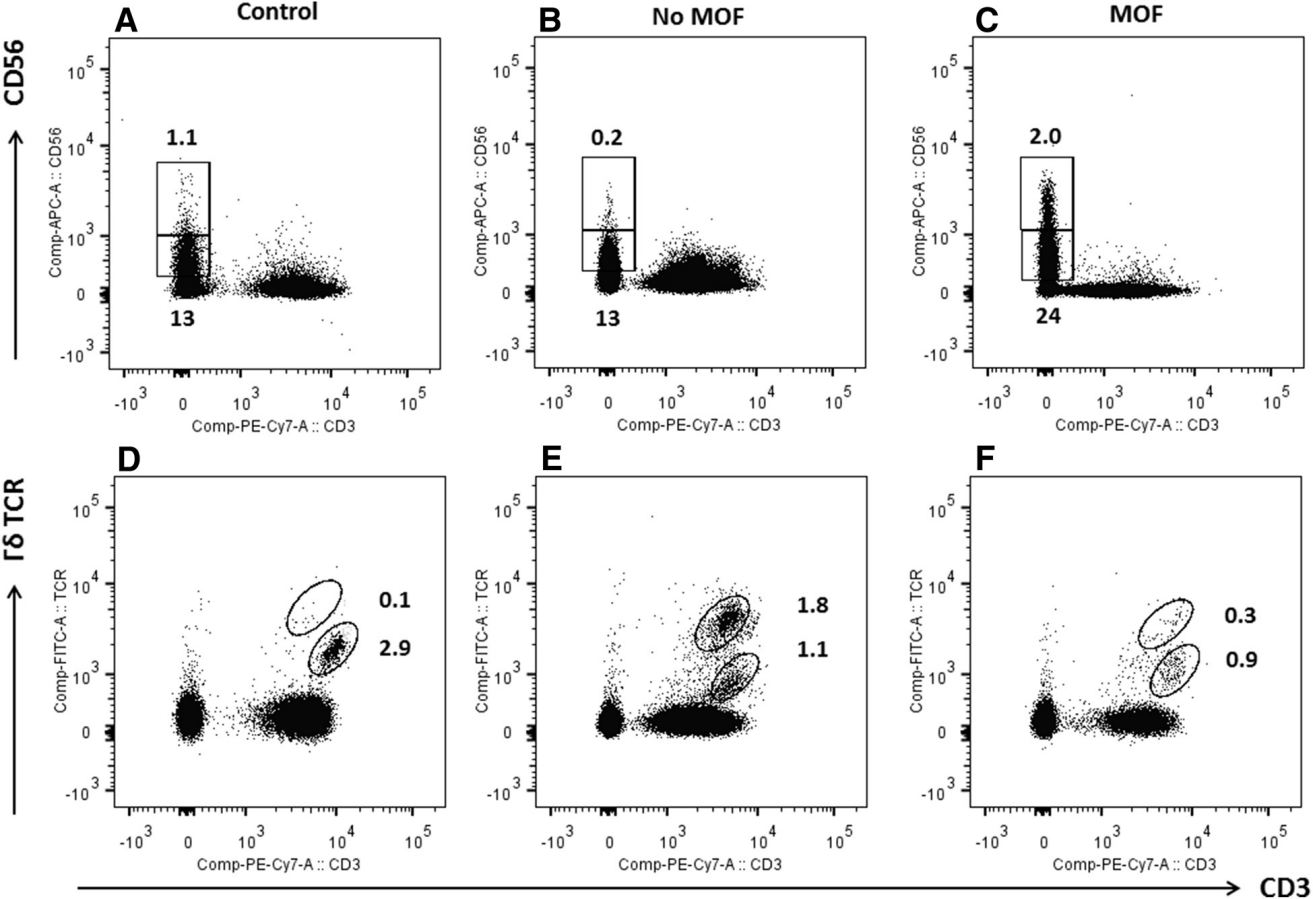
Typical flow cytometry plots of control, no MODS and MODS patients in blood drawn < 2 h following injury. **a**-**c** demonstrate NK cells dim and bright for CD56 and **d**-**f** demonstrate γδ T cell populations low and high for TCR receptor expression. Values shown are percentages of the lymphocyte population. *MODS* multiple organ dysfunction syndrome

**Fig. 2:**
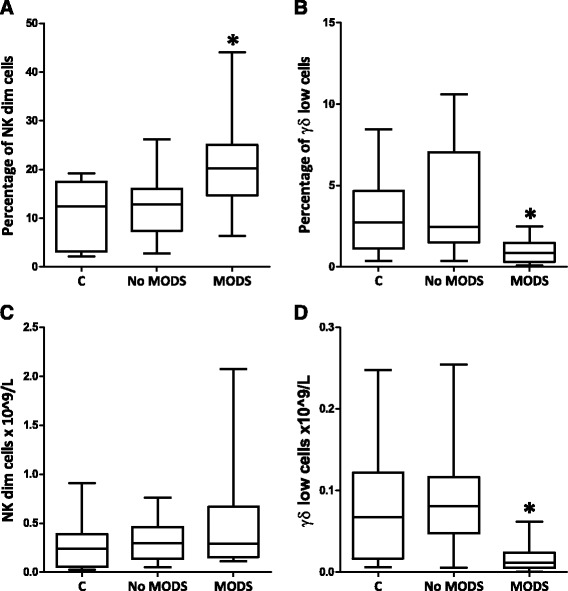
Changes in specific cell populations were associated with the development of MODS. **a** and **c** An elevated percentage of NK dim cells was observed in patients who later developed MODS. **b** and **d** A reduced percentage population and absolute number of γδ-low T lymphocytes was observed in patients who later developed MODS (Table 1). Data are presented as geometric mean (95 % CI), ‘*C*’ indicates the control population and ^*^ denotes *p* < 0.05 when comparing MODS with no MODS using Student’s *t* test. *MODS* multiple organ dysfunction syndrome, *NK* natural killer

Forty patients were sequentially enrolled for flow cytometry analysis. No patient received blood products or more than 500 ml of CSL prior to blood draw. The study cohort included a control group (*n* = 9) and an injured group (*n* = 31) (Table 1). Two patients died within 48 h of injury and were excluded from the analysis. After exclusion of dead cells and doublets, lymphocytes were gated. Several lymphocyte subsets were examined including: T helpers (CD3+ CD4+), cytotoxic T cells (CD3+ CD8+), γδ T cells (CD3+ TCRγδ+) and natural killer (NK) cells (CD3- CD56+) (Table 1, Fig. 1). Within 2 h of injury, patients who later developed MODS had a higher proportion of NK cells in their peripheral blood (MODS vs no MODS: 23 % vs 14 %, *p* < 0.01 (Table 1). This increase was specifically attributable to a rise in the NK dim cell population (MODS vs no MODS: 22 % vs 13 %, *p* < 0.01) with no significant change in NK bright cells (Table 1, Fig. 2). Patients with a NK dim cell population above 15 % at 2 h from injury were almost six times more likely to develop MODS (OR 5.7 95 % CI (1.2–26.3), *p* = 0.03) [14, 15]. The population of γδ-low T lymphocytes was also significantly smaller in patients who subsequently developed MODS (MODS vs no MODS: 0.02 vs 0.09 × 10^9^/L, *p* < 0.01). Early activation of all cells was examined using CD69 but no increased expression was observed (data not shown). Changes in the lymphocyte subpopulations, within 2 h of injury, were associated with later development of MODS.

**Table 1:** Demographics of flow cytometry cohort

Demographics	Controls	No MODS	MODS	*p* value
n	8	19	11	-
% male	100	83	77	0.38
Age^‡^	32 (26–35)	35 (27–44)	34 (27–52)	0.71
ISS^‡^	1 (1–2)	10 (9–18)	33 (25–50)	<0.01
Base deficit (mmol/L)^‡^	−1.9 (−2.3 to −1.0)	−0.5 (−1.4 to −1.6)	3.8 (2.4–7.9)	<0.01
Time of sample from injury (mins)^‡^	66 (60–78)	67 (54–75)	85 (70–95)	0.09
CSL pre-draw (ml)^‡^	0 (0)	0 (0)	500 (0–500)	<0.01
SBP on arrival (mmHg)^‡^	140 (135–149)	139 (131–145)	109 (96–125)	<0.01
Hospital length of stay (days)^‡^	2 (1–3)	7 (3–14)	20 (8–39)	0.07
ICU length of stay (days)^‡^	0 (0)	0 (0)	8 (2–13)	<0.01
28-day mortality % (n)	0 (0)	0 (0)	31	<0.01
Percentage^†^				
Lymphocytes	18 (12–24)	18 (12–23)	17 (8–27)	0.95
Total NK cells	16 (7–24)	14 (11–17)	23 (16–29)	0.02
NK bright	0.6 (0.2–1.0)	1.3 (0.4–2.2)	0.8 (0.4–1.1)	0.12
NK dim	15 (7–23)	13 (10–16)	22 (16–28)	<0.01
Gamma delta high	0.7 (0.4–1.0)	1.4 (0.2–2.5)	1.8 (0.0–3.9)	0.86
Gamma delta low	2.1 (1.2–2.9)	4.0 (2.5–5.5)	0.9 (0.5–1.4)	<0.01
T helpers	32 (26–38)	32 (28–36)	35 (29–41)	0.28
Cytotoxic T cells	23 (18–27)	28 (23–33)	21 (17–25)	0.16
Cell count × 10^9^/L^†^				
Lymphocytes	2.0 (1.3–3.2)	1.4 (0.4–5.1)	2.5 (1.7–3.6)	0.80
Total NK cells	0.2 (0.1–0.6)	0.5 (0.2–0.7)	0.6 (0.2–0.9)	0.14
NK bright	0.01 (0.01–0.02)	0.03 (0.01–0.05)	0.02 (0.01–0.03)	0.20
NK dim	0.2 (0.1–0.5)	0.4 (0.2–0.6)	0.5 (0.2–0.8)	0.12
Gamma delta high	0.01 (0.01–0.03)	0.04 (0.00–0.09)	0.06 (0.00–0.16)	0.73
Gamma delta low	0.03 (0.01–0.08)	0.09 (0.06–0.12)	0.02 (0.01–0.03)	<0.01
T helpers	0.6 (0.4–0.8)	0.9 (0.7–1.1)	0.7 (0.5–0.9)	0.08
Cytotoxic T cells	0.4 (0.3–0.7)	0.8 (0.6–1.0)	0.5 (0.3–0.7)	0.46

*p* values compare no MODS vs MODS, using Student’s *t* test

*MODS* multiple organ dysfunction syndrome, *ISS* Injury Severity Score, *CSL* intravenous crystalloid fluid, *SBP* systolic blood pressure, *ICU* intensive care unit, *NK* natural killer

^‡^Median (interquartile range)

^†^Mean (95 % confidence interval)

### Cytokine levels indicate early innate lymphocyte activation

**Fig. 3:**
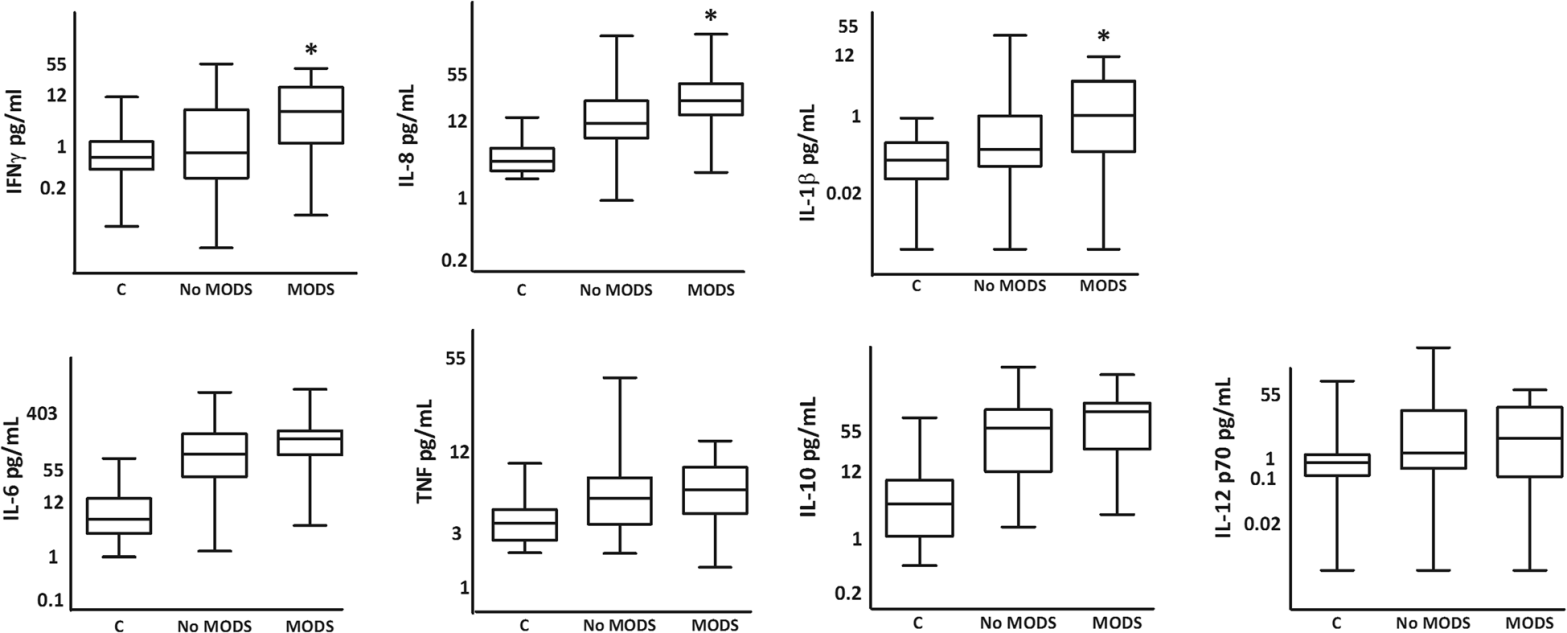
Cytokine concentrations demonstrate early activation of lymphocytes following critical injury. Cytokines were quantified in plasma drawn at < 2 h following injury in trauma patients (*n* = 87) with critical injury severity (ISS ≥ 25) and control patients (*n* = 39). Patients who developed MODS (*n* = 27) had higher concentrations of IFN-γ, IL-1β and IL-8 when compared to critically injured patients who did not develop MODS (*n* = 60). IFN-γ: 0.6 (0.4–0.9), 1.0 (0.6–1.8), 4.1 (1.8–9.0), *p* = 0.01. TNF-α: 3.3 (2.9–3.7), 5.2 (4.5–6.1), 5.7 (4.6–7.0), *p* = 0.54. IL-1β: 0.1 (0.0–0.1), 0.2 (0.1–0.4), 0.7 (0.3–1.9), *p* = 0.04. IL-6: 6.1 (4.2–8.8), 91.0 (60.8–136.2), 176.6 (106.1–294.1), *p* = 0.06. IL-8: 3.8 (3.2–4.5), 12 (9.1–15.9), 21.5 (14.8–31.2), *p* = 0.02. IL-10: 3.6 (2.5–5.3), 41.4 (28.0–61.2), 66.2 (40.4–108.5), *p* = 0.17. IL-12 p70: 0.6 (0.3–1.1), 2.2 (1.2–3.9), 2.5 (0.9–6.8), *p* = 0.78. Data are presented as C, no MODS, MODS, *p* value in pg/mL as geometric mean (95 % CI). ^*^Denotes *p* < 0.05 using Student’s *t* test comparing no MODS and MODS. *IFN-γ* interferon gamma, *IL* interleukin, *MODS* multiple organ dysfunction syndrome, *TNF-α* tumour necrosis factor alpha

A cohort of blunt polytrauma patients with an ISS ≥ 25 was identified from the ACIT II database and divided by outcome (MODS *n* = 27, no MODS *n* = 60). No patient had blood products or more than 500 ml of crystalloid before blood draw. The two groups had comparable levels of injury severity and shock (MODS vs no MODS: ISS = 34 (29–39) vs 30 (27–38), *p* = 0.49; BD = 9 (2.5–9.7) vs 3.1 (1.8–6.7) mmol/L, *p* = 0.06). Cytokine quantification was performed on stored plasma from these patients. All seven cytokines were elevated above control levels, confirming systemic activation of inflammation in both groups. Comparison between the two groups demonstrated higher concentrations of interferon gamma (IFN-γ), interleukin (IL)-1β and IL-8 in patients who developed MODS (Fig. 3). Despite matched injury characteristics, patients who developed MODS displayed a specific pattern of increased inflammation at 2 h following injury.

### Lymphopenia is associated with early innate lymphocyte aberrations, MODS and mortality

**Fig. 4:**
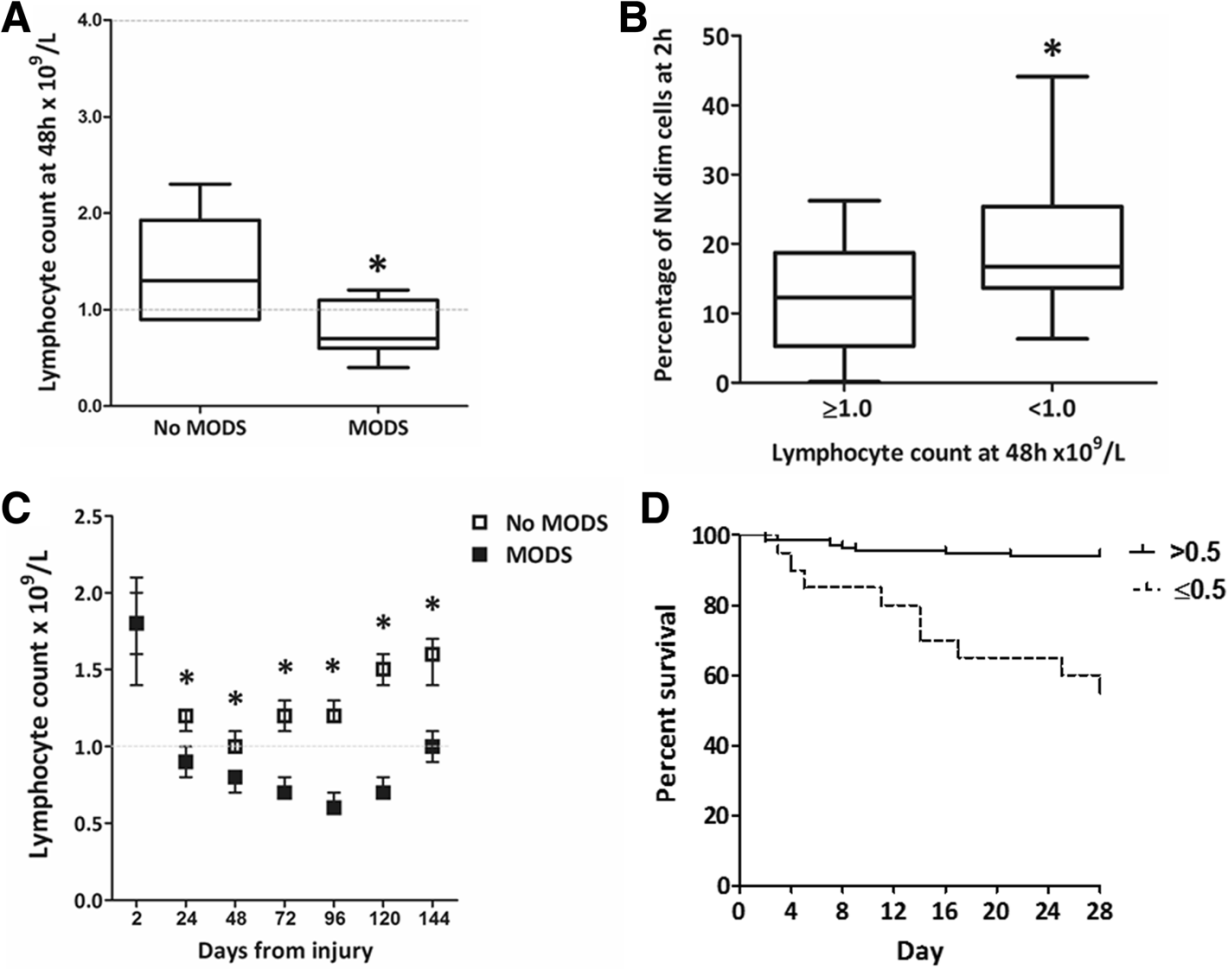
Lymphopenia at 48 h was associated with MODS, high NK dim populations at admission and increased mortality. **a** Patients who developed MODS (*n* = 11) were found to have a lower lymphocyte count at 48 h compared to those who did not develop MODS (*n* = 19). No MODS = 1.3 (1.0–1.7) × 10^9/L, MODS = 0.8 (0.7–1.1) × 10^9/L, *p* = 0.01. Data are presented as median (IQR) and ^*^ denotes *p* < 0.05 using Mann-Whitney *U* test. **b** Patients with lymphopenia at 48 h had a higher percentage of NK dim cells in their circulation at 2 h post injury (*n* = 31). Lymphocyte count ≥ 1.0 = NK dim 12 % (6–18), < 1.0 = NK dim 17 % (14–25), *p* < 0.01. Data are presented as median (IQR) and ^*^ denotes *p* < 0.05 using Mann-Whitney *U* test. **c** MODS patients had persistent lymphopenia throughout the first 6 days of admission. Daily lymphocyte count was examined in a cohort of ICU patients with an ISS > 15 after blunt trauma (*n* = 280). All patients had a normal lymphocyte count at admission. Patients who developed MODS had lymphocyte counts well below the normal range from 24 h to 120 h (D6). No MODS: 1.8 (1.6–2.0), 1.2 (1.1–1.2), 1.0 (1.0–1.1), 1.2 (1.1–1.3), 1.2 (1.2–1.3), 1.5 (1.4–1.6), 1.6 (1.4–1.7). MODS: 1.8 (1.4–2.1), 0.9 (0.8–1.0), 0.8 (0.7–0.8), 0.7 (0.7–0.8), 0.6 (0.6–0.7), 0.7 (0.7–0.8), 1.0 (0.9–1.1). Data are presented as mean (95 % CI), *dotted line* indicates the normal range. Statistical significance was tested using a two-way mixed ANOVA on natural log data (*p* < 0.001). This was supported by testing for simple main effects using a general linear model univariate analysis at each time point (2 h: *p* = 0.21, 24 h–144 h: *p* < 0.001, denoted by^*^). Data are presented in order 2 h–144 h: F (1278) = 1.65, 22.91, 30.90, 92.59, 202.84, 196.43, 48.74 and η^2^ = < 0.01, 0.08, 0.10, 0.25, 0.42, 0.41, 0.15. **d** Severe lymphopenia at 48 h was associated with a high mortality rate. Patients with a lymphocyte count ≤ 0.5 × 10^9^/L had a 45 % mortality rate compared with 6 % in those with a count > 0.5 × 10^9^/L, *p* < 0.001 using the Mantel-Cox test (*n* = 280). *MODS* multiple organ dysfunction syndrome, *NK* natural killer

Lymphocyte count during the first 7 days of admission was examined in the flow cytometry cohort (*n* = 31). Patients who developed MODS were found to have a lower lymphocyte count at 48 h than those who recovered without MODS (Fig. 4a). Patients who developed lymphopenia by 48 h (count below 1.0 × 10^9^/L) had higher NK dim populations at admission compared with those who maintained their lymphocyte count (Fig. 4b). Lymphocyte count during the first 7 days was then examined in a larger cohort. All patients in this cohort (*n* = 280) were severely injured and admitted to the ICU, 33 % of these fulfilled the study definition of MODS (Table 2). Patients in the MODS group, had elevated SOFA scores at admission, median (interquartile range [IQR]) of 10 (8–11) and scores remained high throughout the first 96 h. Patients in the no MODS group had elevated SOFA scores at admission median 9 (8–10) but rapidly recovered. All patients had a normal lymphocyte count within 2 h of injury. By 24 h, the mean lymphocyte count had fallen below the normal range in the MODS group (Fig. 4c). The difference was even more pronounced at 48 h and continued to day 7. Lymphopenia, neutrophil, monocyte, eosinophil and basophil counts were also examined but no difference between MODS and no MODS patients was observed. Binary logistic regression analysis demonstrated that lymphocyte count at 48 h, ISS and BD were independent predictors of MODS development, lymphocyte count at 48 h being the strongest (Table 3). Patients with lymphopenia at 48 h, had a higher mortality rate than those with a normal count (17 % vs 5 % *p* = 0.02). Furthermore, patients with severe lymphopenia at 48 h (lymphocyte count ≤ 0.5 × 10^9^/L) had a mortality rate of 45 % compared with only 6 % in those with a count above 0.5 × 10^9^/L (Fig. 4d).

**Table 2:** Demographics of ICU cohort

Demographics	No MODS	MODS	*p* value
n	187 (67)	93 (33)	-
% male	147 (79)	76 (83)	0.63
Age^‡^	39 (25–57)	39 (26–51)	0.36
ISS^‡^	26 (22–34)	34 (26–41)	<0.01
Base deficit (mmol/L)^‡^	3.7 (1.3–7.3)	4.7 (2.4–8.4)	0.02
SBP on arrival (mmHg)‡	126 (98–144)	122 (86–147)	0.16
PRBC (units)^‡^	2 (0–6)	3 (0–8)	0.42
SOFA on admission^‡^	9 (8–10)	10 (8–11)	0.03
Hospital length of stay (days)^‡^	25 (14–38)	33 (24–50)	<0.01
ICU length of stay (days)^‡^	8 (5–12)	16 (10–23)	<0.01
Infection %	76 (41)	61 (66)	<0.01
28-day mortality %	15 (8)	9 (10)	0.65

*p* values compare no MODS vs MODS, using Mann-Whitney *U* test

*MODS* multiple organ dysfunction syndrome, *ICU* intensive care unit, *ISS* Injury Severity Score, *SBP* systolic blood pressure, *PRBC* packed red blood cells administered in first 24 h, *SOFA* Sequential Organ Failure Score

^‡^Median (interquartile range)

**Table 3:** Binary logistic regression analysis of variables independently associated with the development of MODS

Variable	Univariate	B (SE)	Odds ratio (95 % CI)	*p* value
Included				
LC 48 h	<0.01	−2.05 (0.47)	0.13 (0.05–0.32)	<0.01
BD	<0.01	0.07 (0.03)	1.07 (1.01–1.13)	0.01
ISS	<0.01	0.02 (0.01)	1.02 (1.00–1.05)	0.05
Constant	-	−0.00 (0.56)	-	-
Excluded				
24 h PRBC	0.07			
Age	0.34			
Gender	0.39			
SBP	0.82			

CI: confidence intervals, Hosmer and Lemeshow test = 0.75, Cox and Snell R^2^ = 0.15, Nagelkerke R^2^ = 0.21, model chi-sq = 44.78, *p* < 0.01

*MODS* multiple organ dysfunction syndrome, *LC* lymphocyte count 109/L, *BD* base deficit, *ISS* Injury Severity Score, *PRBC* packed red blood cells administered in first 24 h, *SBP* systolic blood pressure

## Discussion

This study has examined circulating lymphocytes in severely injured trauma patients and provides new evidence to suggest that lymphocyte activity within the first 2 h following injury is related to the development of MODS and lymphopenia. High NK dim and low γδ-low T lymphocyte populations coupled with high IFN-γ concentrations suggest that lymphocytes are active in the immediate post-injury phase. The association between high NK dim cells at admission and lymphopenia at 48 h links early changes to later immune incompetence and a strong association between lymphopenia and the development of MODS has been demonstrated for the first time. Taken together, these findings implicate lymphocytes in the pathogenesis of MODS and suggest that immunological events activated prior to hospital admission may influence recovery.

Natural killer (NK) cells account for 10–15 % of the circulating lymphocytes in humans and are fundamental to the ‘innate’ immune response [15]. The logistical challenges of conducting trauma research mean that very few studies have specifically examined immune cell populations after injury and none have focused on the first 2 h. NK cell populations have previously been shown to decrease within 24 h and later functional impairment has also been described [28, 29]. The mechanisms behind our observed rise immediately after trauma remain unclear but NK cells are known to increase in circulation in response to drivers such as catecholamines [30]. This may reflect rapid mobilisation from bone marrow and other secondary lymphoid tissues, or expedited maturation from bright to dim CD56 expression [14]. The NK dim cell subset are considered to have a ‘cytotoxic’ phenotype, but their functional role during the early post-injury response remains unclear [15, 31]. The IFN-γ measured at 2 h is presumed to originate from NK cells as they store preformed IFN-γ which can be rapidly released after activation [32].

In contrast, γδ-low T lymphocytes are predominantly tissue-resident and make up < 5 % of the circulating T cell population in humans [33]. Their presence in blood, likely reflects migration. They are regarded as cytoprotective, therefore the association between low concentrations of these cells and the development of MODS is novel and may be important [13, 34]. Only one previous study has examined γδ T cells in human trauma patients (*n* = 7); this reported a fall around 72 h, which was attributed to apoptosis [35]. More recently, a murine study has demonstrated that γδ T cells regulate immune cell infiltration of lung tissue, which may influence acute lung injury [36]. There are large gaps in our understanding of the immediate post-injury cellular immune response and detailed immunological studies at this pivotal time period are required.

Lymphopenia after trauma has been associated with mortality but the association with MODS is a new finding. Although ISS and BD are highlighted in the regression analysis as key risk factors for MODS development, the 48-h lymphocyte count is the strongest predictor of the included variables. In addition, patients with a very low lymphocyte count ≤ 0.5 × 10^9^/L at 48 h have a mortality rate of 45 %. This suggests that lymphocyte-related events within the first 48 h are critical to recovery. Lymphopenia is widely attributed to infection-induced apoptosis but this study challenges that assumption as lymphopenia developed within 24 h, several days before the onset of clinical infection [19, 37–40]. Although not currently considered to be of clinical relevance, this study suggests that lymphocyte count at 48 h could be an early indicator of poor prognosis. The findings strengthen the evidence that lymphopenia may be involved in the pathogenesis of adverse outcome and that restoration of lymphocyte count may be essential to recovery [19].

Several limitations of this study are acknowledged, principally that sequential experiments complicate data interpretation and a single patient cohort would have been preferable. The flow cytometry cohort was small (*n* = 40) and patients with worse outcomes were more severely injured. Some of the observations may therefore reflect injury severity. This is a common critique of trauma research and future work will endeavour to obtain larger, matched injury groups. In addition, although a high percentage of NK dim cells was observed, the absolute count was not significantly different and this is also attributed to the small size of the study. MODS is currently defined using organ scores; in accordance with the study definition, MODS was not formally diagnosed until after 96 h although SOFA scores were elevated from admission. We acknowledge that it is impossible to identify the precise onset of MODS using organ scores. The 7-day lymphocyte data are influenced by survivor bias. Finally, despite transformation, the lymphocyte count data violated key assumptions required for a valid two-way mixed ANOVA test. This was due to the stark differences between the 2 h samples and subsequent days. Significance was confirmed with valid tests at each time point but this limitation is accepted.

## Conclusions

This study has demonstrated that early lymphocyte activity is related to the development of MODS and lymphopenia. The observed increase in NK dim cells, reduction in δγ-low T cells and high IFN-γ concentration at 2 h after injury, suggest that lymphocytes may be more important to the immediate post-injury response than previously appreciated. Development of MODS appears to be influenced by cellular events which are initiated prior to hospital arrival and orchestrated within the first 48 h. The study highlights the need for detailed examination of cellular responses in trauma patients, particularly in the first few hours, and emphasises the importance of well-characterised patient cohorts and consistent sampling time points in order to characterise this complex, dynamic response. The study opens up new lines of investigation for trauma research and suggests that there may be new opportunities for intervention to expedite recovery.

## Key messages

- Lymphocyte activity within 2 h of injury is associated with the development of MODS and lymphopenia after trauma.
- Development of MODS is associated with high NK dim cells and low γδ-low T lymphocyte populations at admission.
- Lymphopenia occurs within 24 h after severe injury and persists in patients with MODS
- Patients with a lymphocyte count ≤ 0.5 × 10^9^/L at 48 h had a 45 % mortality rate

## Abbreviations

BD: base deficit; CSL: compound sodium lactate; DAMPs: damage-associated molecular patterns; DCR: damage control resuscitation; ICU: intensive care unit; IFN-γ: interferon gamma; IL: interleukin; ISS: Injury Severity Score; MODS: multiple organ dysfunction syndrome; MTC: major trauma centre; NK: natural killer; SOFA: Sequential Organ Failure Score
